# The In Vitro Antibiotic Susceptibility of Malaysian Isolates of *Burkholderia pseudomallei*

**DOI:** 10.1155/2013/121845

**Published:** 2013-09-26

**Authors:** Norazah Ahmad, Rohaidah Hashim, Azura Mohd Noor

**Affiliations:** Bacteriology Unit, Institute for Medical Research, Jalan Pahang, 50588 Kuala Lumpur, Malaysia

## Abstract

Acute melioidosis may present as localised or septicaemic infections and can be fatal if left untreated. *Burkholderia pseudomallei* resistant to antibiotics used for the treatment of melioidosis had been reported. The aim of this study was to determine the in vitro antibiotic susceptibility patterns of *Burkholderia pseudomallei* isolated in Malaysia to a panel of antibiotics used for the treatment of melioidosis and also to potential alternative antibiotics such as tigecycline, ampicillin/sulbactam, and piperacillin/tazobactam. A total of 170 *Burkholderia pseudomallei* isolates were subjected to minimum inhibitory concentration determination using *E*-test method to eleven antibiotics. All isolates were sensitive to meropenem and piperacillin/tazobactam. For ceftazidime, imipenem, amoxicillin/clavulanic acid, and doxycycline resistance was observed in 1 isolate (0.6%) for each of the antibiotics. Trimethoprim/sulfamethoxazole resistance was observed in 17 (10%) isolates. For other antibiotics, ampicillin/sulbactam, chloramphenicol, tigecycline, and ciprofloxacin resistance were observed in 1 (0.6%), 6 (3.5%), 60 (35.3%) and 98 (57.7%) isolates respectively. One isolate B170/06 exhibited resistance to 4 antibiotics, namely, ciprofloxacin, chloramphenicol, trimethoprim/sulfamethoxazole, and tigecycline. In conclusion, the Malaysian isolates were highly susceptible to the current antibiotics used in the treatment of melioidosis in Malaysia. Multiple resistances to the antibiotics used in the maintenance therapy are the cause for a concern.

## 1. Introduction

The causative agent of melioidosis, *Burkholderia pseudomallei*, is endemic in the Northern part of Australia and Southeast Asia including Malaysia. Acute melioidosis may present as localized or septicaemic infections and can be fatal if left untreated. It was the common cause of community-acquired pneumonia in Northeastern Thailand and was attributed as the cause of fatal community-acquired bacteremic pneumonia in Northern Australia [1, 2]. Latent infection may remain asymptomatic for years only to be reactivated from a latent focus when the host is immunocompromised. Therefore, it is important to treat melioidosis with prolonged course of antibiotics so as to avoid disease relapses which are commonly associated with short courses of antibiotics. Some patients may default treatment or take improper dosage of antibiotics because of the long duration of treatment, and this may contribute to the relapse or the development of resistance. 

*Burkholderia pseudomallei* is intrinsically resistant to a wide range of antibiotics which include some *β*-lactam antibiotics, aminoglycosides, and macrolides. The antibiotics that are currently being used for the therapy of melioidosis are ceftazidime, imipenem, meropenem, amoxicillin/clavulanate, cefoperazone/sulbactam, trimethoprim-sulfamethoxazole (TMP-SMX), doxycycline, and chloramphenicol. The antibiotic regime for the treatment of melioidosis varies from one country to another. In Malaysia, ceftazidime alone, or in combination with TMP-SMX or cefoperazone/sulbactam alone or in combination with TMP-SMX or imipenem, is the recommended antibiotic of choice for the intensive phase of treatment followed by oral TMP-SMX plus doxycycline or amoxicillin/clavulanate in the maintenance phase [3]. The development of resistance of *Burkholderia pseudomallei* to some of these antibiotics has been reported in neighbouring countries such as Singapore and Thailand [4, 5]. Reports on the antibiotic susceptibility of *Burkholderia pseudomallei* in Malaysia have been limited to a few selected antibiotics and a smaller number of tested strains and from restricted demographic areas [6, 7]. 

Therefore, the aim of the study was to determine the in vitro antibiotic susceptibility patterns of clinical isolates of *Burkholderia pseudomallei* to a panel of antibiotics used for the treatment of melioidosis and also to the potential alternative antibiotics such as tigecycline, ampicillin/sulbactam, and piperacillin/tazobactam. 

## 2. Materials and Methods

### 2.1. Bacterial Isolates

A total of 170 *Burkholderia pseudomallei* nonrepeat clinical isolates were collected from the year 2001 until the year 2009, from the microbiology laboratories of 29 government hospitals situated in 11 out of 14 states in Malaysia (Table 1). These isolates were sent from these hospitals for the confirmation of identification at the Bacteriology Unit, Institute for Medical Research. Species identification was carried out by Gram-staining, motility, API 20NE (bioMèrieux), and polymerase chain reaction technique using specific 16rRNA primers as described by Brook et al. 1997 [8]. The strains were stored at −80°C in 20% glycerol and were revived by subculturing onto blood agar plates before being further used.

### 2.2. Antibiotic Susceptibility Testing

Minimum inhibitory concentrations (MIC) of the antibiotics were determined by *E*-test (bioMèrieux) following the manufacturer's instructions. Eleven antibiotics were tested, namely, ceftazidime, imipenem, meropenem, amoxicillin/clavulanic acid, TMP-SMX, ciprofloxacin, doxycycline, chloramphenicol, piperacillin/tazobactam, ampicillin/sulbactam, and tigecycline. 

A 0.5 McFarland suspension was made for each bacterial isolates and then inoculated on Mueller-Hinton (MH) (BD) agar. The *E*-test strips of each antibiotic were placed on the MH agar and incubated overnight at 37°C. The zones of inhibition were noted after 18 hours of incubation. The MIC (*μ*g/mL) interpretation for susceptible (*s*), intermediate (*i*), and resistant (*r*) for amoxicillin-clavulanic acid (*s* ≤ 8/4, *i*  16/8, *r* ≥ 32/16), ceftazidime (*s* ≤ 8, *i*  16, *r* ≥ 32), imipenem (*s* ≤ 4, *i*  8, *r* ≥ 16), doxycycline (*s* ≤ 4, *i*  8, *r* ≥ 16), and TMP-SMX (*s* ≤ 2/38, -, *r* ≥ 4/76) was carried out following the CLSI approved guideline M45-A2 [9]. For ciprofloxacin (*s* ≤ 1, *i*  2, *r* ≥ 4), chloramphenicol (*s* ≤ 8, *i*  16, *r* ≥ 32), piperacillin/tazobactam (*s* ≤ 16/4, *i*  32/4–64/4, *r* ≥ 128/4), and ampicillin/sulbactam (*s* ≤ 8/4, *i*  16/8, *r* ≥ 32/16), the MIC (*μ*g/mL) for Enterobacteriaceae was referred [10]. For tigecycline, the US FDA approved breakpoints for Enterobacteriaceae were used (*s* ≤ 2, *i*  4, *r* ≥ 8). For meropenem, the interpretative criteria outlined by the *E*-test manufacturer for aerobes were followed (*s* ≤ 4, *i*  8, *r* ≥ 16). Any values which were in between the sensitive and resistant breakpoints were interpreted as intermediates. *Escherichia coli* ATCC 25922 and *Pseudomonas aeruginosa* ATCC 27853 were used as controls.

## 3. Results

All strains were sensitive to meropenem and piperacillin/tazobactam. Sensitivity to ceftazidime, imipenem, amoxicillin/clavulanic acid, ampicillin/sulbactam, and doxycycline was noted in 169 (99.4)% of the isolates. One isolate was shown to have heterogenous population with ceftazidime susceptibility of 8 *μ*g/mL and ceftazidime resistance of ≥256 *μ*g/mL. This strain, however, remained susceptible to other antibiotics. The isolate that was intermediately resistant to imipenem (MIC 6 *μ*g/mL) was also resistant to amoxicillin/clavulanic acid and ampicillin/sulbactam. For chloramphenicol and TMP-SMX, 164 (96.5%) and 153 (90%) of the strains were susceptible, respectively. Only 72 (42.3%) isolates were susceptible to ciprofloxacin while 94 (55.3%) isolates showed intermediate resistance with MIC of 1.5–3.0 *μ*g/mL. Susceptibility to tigecycline was observed in 110 isolates (64.7%) while intermediate resistance was noted in 59 (34.7%) of the isolates. One isolate was resistant to tigecycline at the MIC of 8.0 *μ*g/mL.

The minimum concentration of antibiotics that inhibited 50% (MIC_50_) and 90% (MIC_90_) of the isolates and the range of MICs of the tested antibiotics were shown in Table 2. The MIC_90_ of most of the antibiotics were within the range of 0.38 to 4.0 *μ*g/mL. Chloramphenicol was shown to have higher MIC_50_ and MIC_90_ values than the other antibiotics (MIC_50_, 6.0 *μ*g/mL; MIC_90_ 8.0 *μ*g/mL). Imipenem (MIC_50,_ 0.38 *μ*g/mL; MIC_90_, 0.38 *μ*g/mL) was more active towards these strains than meropenem (MIC_50_, 0.75 *μ*g/mL and MIC_90_, 1.0 *μ*g/mL).

The resistance pattern observed among these strains was mainly monoresistance where 7 strains showed resistance to a single antibiotic, either to ceftazidime (1 strain), ciprofloxacin (1 strain), or trimethoprim/sulfamethoxazole (5 strains). Two strains showed resistance to 2 different types of antibiotics (1 isolate to ciprofloxacin & trimethoprim/sulfamethoxazole; 1 strain to amoxicillin/clavulanic acid and ampicillin/sulbactam). One strain showed multiple resistances to 4 antibiotics namely ciprofloxacin, chloramphenicol, trimethoprim/sulfamethoxazole, and tigecycline and intermediate resistance to doxycycline. 

## 4. Discussion

This study showed that the local strains were highly susceptible to meropenem and piperacillin/tazobactam. For ceftazidime, imipenem, amoxicillin/clavulanic acid, and doxycycline, only 0.6% of the isolate was resistant to these antibiotics. A highly resistant subpopulation of ceftazidime resistance was detected in one of the strain. This was a blood isolate from a patient who had no past record of melioidosis and had passed away a day after admission before the culture result was obtained. Primary resistance to ceftazidime is rare and studies had documented that the development of resistance emerge mainly during treatment [11, 12]. 

The susceptibility to trimethoprim/sulfamethoxazole is slightly lower compared to 97.5% in the another local study on 80 *Burkholderia pseudomallei* [6]. Only 4.0% of our isolates was resistant to trimethoprim/sulfamethoxazole compared to 13% resistance rate in Thailand [13]. Our local strains were also highly susceptible to chloramphenicol. An open label study in Thailand had shown that the combination of chloramphenicol, TMP-SMX, and doxycycline was associated with higher adverse effect compared to TMP-SMX and doxycycline only [14]. Chloramphenicol was not included in the maintenance therapy of melioidosis in Malaysia [3].

The antibiotic susceptibility rate of ciprofloxacin was low at 42.3%, and 90% of the isolates were inhibited at the intermediate MIC value of 2 *μ*g/mL, which implied that ciprofloxacin was less effective towards these strains. Fluoroquinolones have been shown to be less effective clinically [15, 16]. 

A study on the activity of tigecycline against *Burkholderia pseudomallei* in Thailand showed low MIC_50_ and MIC_90_ (0.5 *μ*g/mL and 2.0 *μ*g/mL) [17]. This finding is similar to another study in Malaysia [7]. In contrast, the isolates tested in this study were less susceptible to tigecycline, where higher MIC_50_ and MIC_90_ values (0.75 *μ*g/mL and 4.0 *μ*g/mL) were observed. The susceptibility of tigecycline was only 64.7%, and 34.7% of the strains were inhibited at the intermediate range of MIC of 3.0–6.0 *μ*g/mL. This is in concordance with another study in Australia, where 85.5% of the isolates were inhibited at an intermediate MIC of 4.0 *μ*g/mL with lower susceptibility rate of 14.5% [18]. 

The isolate that was intermediately resistant to imipenem with MIC 6 *μ*g/mL, also had co-resistance to amoxicillin/clavulanic acid and ampicillin/sulbactam. This isolate was susceptible to meropenem at MIC 1.5 *μ*g/mL. The MIC_50_ and MIC_90_ of meropenem were noted to be higher than those in imipenem; however, meropenem is still effective against all the strains tested. In Malaysia, meropenem or imipenem has been used for severe infection or in the event of treatment failure with ceftazidime. All the multiply resistant strains were still susceptible to ceftazidime but some of these strains were resistant to the antibiotics used in the maintenance therapy. The mechanism of resistance of these isolates will be further studied.

In conclusion, the *Burkholderia pseudomallei* isolates from Malaysia were highly susceptible to the antibiotics used in the treatment of melioidosis namely ceftazidime, trimethoprim/sulfamethoxazole, imipenem, meropenem, amoxicillin-clavulanate, and doxycycline. Ciprofloxacin and tigecycline were not active in vitro against these isolates. The presence of isolates that were resistant to the antibiotics used for maintenance therapy is of concern because this could affect the treatment outcome and may lead to the relapse of infection. 

## Figures and Tables

**Table 1:** The information on the strains used in this study.

Lab ID	Year isolated	State	Sex	Source
RZ 14/01	2001	Negeri Sembilan	m	wound swab
RZ 16/01	2001	Negeri Sembilan	m	urine
RZ 21/01	2001	Perak	f	blood
RZ 31/01	2001	Selangor	f	NA
RZ 37/01	2001	Perak	m	sputum
RZ 116/01	2001	Kedah	m	pus
RZ 166/01	2001	Perak	m	blood
RZ 169/01	2001	Perak	m	blood
RZ 191/01	2001	Perak	f	blood
RZ 12/02	2002	Negeri Sembilan	f	pus
RZ 50/02	2002	Johor	m	blood
RZ 51/02	2002	Johor	m	swab
RZ 69/02	2002	Perak	m	blood
RZ 94/02	2002	Perak	m	blood
RZ 95/02	2002	Perak	m	blood
RZ 107/02	2002	Perak	m	blood
RZ 130/02	2002	Sarawak	m	blood
RZ 143/02	2002	Perak	m	blood
RZ 144/02	2002	Perak	m	blood
RZ 145/02	2002	Perak	m	blood
RZ 176/02	2002	Selangor	f	blood
RZ 194/02	2002	Pahang	f	blood
RZ 203/02	2002	Perak	m	blood
RZ 207/02	2002	Penang	m	pus
RZ 15/03	2003	Perak	m	urine
RZ 51/03	2003	Perak	f	blood
RZ 58/03	2003	Penang	f	blood
RZ 76/03	2003	Penang	m	blood
RZ 4/05	2005	Johor	m	NA
RZ 5/05	2005	Pahang	m	NA
RZ 7/05	2005	Pahang	f	NA
RZ 11/05	2005	Sarawak	m	NA
RZ 14/05	2005	Sarawak	m	NA
RZ 15/05	2005	Sarawak	m	NA
RZ 18/05	2005	Johor	m	NA
RZ 46/05	2005	Sarawak	m	NA
RZ 49/05	2005	Perlis	m	NA
RZ 50/05	2005	Sarawak	m	NA
RZ 52/05	2005	Perak	f	NA
RZ 61/05	2005	Sabah	f	NA
RZ 76/05	2005	Johor	f	NA
RZ 77/05	2005	Perak	m	NA
RZ 85/05	2005	Johor	m	NA
RZ 86/05	2005	Johor	m	NA
RZ 87/05	2005	Pahang	m	NA
RZ 88/05	2005	Sabah	f	NA
RZ 77/06	2006	Johor	m	blood
RZ 97/06	2006	Selangor	m	blood
RZ 102/06	2006	Johor	m	Blood
RZ 125/06	2006	Johor	m	Blood
B 146/06	2006	Pahang	m	Blood
B 149/06	2006	Pahang	m	Blood
B 150/06	2006	Pahang	m	Pus
B 151/06	2006	Pahang	m	Blood
B 152/06	2006	Pahang	m	Blood
B 153/06	2006	Kedah	m	Blood
B 154/06	2006	Kedah	m	Blood
B 155/06	2006	Kedah	f	Blood
B 156/06	2006	Kedah	f	Pus
B 158/06	2006	Pahang	m	Blood
B 159/06	2006	Pahang	f	Blood
B 160/06	2006	Pahang	m	Blood
B 161/06	2006	Pahang	m	Blood
B 162/06	2006	Pahang	m	Blood
B 164/06	2006	Pahang	m	Blood
B 168/06	2006	Perak	m	Blood
B 169/06	2006	Pahang	m	Blood
B 170/06	2006	Kedah	f	Pus
B 171/06	2006	Kedah	m	Blood
B 172/06	2006	Pahang	m	Blood
B 174/06	2006	Perak	m	Blood
B 175/06	2006	Perak	m	Pus
B 176/06	2006	Perak	m	Urine
B 177/06	2006	Kedah	m	Blood
B 178/06	2006	Kedah	f	Blood
B 179/06	2006	Kedah	m	Aspirate
B 180/06	2006	Kedah	m	Blood
B 181/06	2006	Kedah	m	Blood
B 183/06	2006	Kedah	m	Blood
B 184/06	2006	Kedah	m	Pus
RZ 3/07	2007	Pahang	m	Blood
RZ 7/07	2007	Penang	m	Blood
RZ 8/07	2007	Penang	m	Blood
RZ 9/07	2007	Penang	m	Blood
RZ 19/07	2007	Johor	m	Blood
RZ 57/07	2007	Selangor	f	NA
RZ 162/07	2007	Sarawak	f	Blood
RZ 191/07	2007	Selangor	m	Blood
RZ 355/07	2007	Johor	m	Blood
B 185/07	2007	Kedah	m	Blood
B 186/07	2007	Kedah	m	Blood
B 187/07	2007	Kedah	m	Blood
B 191/07	2007	Penang	m	Blood
B 192/07	2007	Kedah	m	Blood
B 193/07	2007	Selangor	m	NA
B 194/07	2007	Selangor	f	NA
B 195/07	2007	Johor	f	Blood
B 196/07	2007	Johor	m	Blood
B 197/07	2007	Johor	m	Blood
B 198/07	2007	Perak	m	Blood
B 199/07	2007	Perak	m	Blood
B 200/07	2007	Perak	m	Blood
B 201/07	2007	Perak	m	Blood
B 202/07	2007	Johor	m	Blood
RZ 27/08	2008	Sarawak	m	Blood
RZ 61/08	2008	Sarawak	m	Blood
RZ 64/08	2008	Sarawak	m	Blood
RZ 72/08	2008	Selangor	m	Blood
RZ 76/08	2008	Sarawak	m	knee aspirate
RZ 77/08	2008	Sarawak	f	Sputum
RZ 91/08	2008	Selangor	m	Blood
RZ 96/08	2008	Pahang	f	Blood
RZ 107/08	2008	Selangor	f	Peritoneal fluid
RZ 115/08	2008	Sarawak	m	Abscess
RZ 118/08	2008	Sarawak	m	Blood
RZ 120/08	2008	Sarawak	m	Blood
RZ 160/08	2008	Pahang	f	Blood
RZ 179/08	2008	Sarawak	m	Blood
RZ 196/08	2008	Sarawak	m	Urine
RZ 269/08	2008	Johor	f	Blood
RZ 276/08	2008	Sarawak	m	Blood
RZ 299/08	2008	Sarawak	m	Pus
RZ 305/08	2008	Sarawak	m	Blood
RZ 307/08	2008	Johor	m	Tissue
RZ 365/08	2008	Sarawak	m	Blood
HB 1/09	2009	Pahang	m	Abscess
RZ 9/09	2009	Selangor	m	Blood
RZ 43/09	2009	Sarawak	f	Blood
RZ 116/09	2009	Wilayah Persekutuan	f	Blood
RZ 117/09	2009	Wilayah Persekutuan	f	Blood
RZ 118/09	2009	Wilayah Persekutuan	f	Blood
RZ 167/09	2009	Sarawak	m	Urine
RZ 168/09	2009	Sarawak	f	Sputum
RZ 169/09	2009	Sarawak	m	Blood
RZ 170/09	2009	Sarawak	m	Blood
RZ 193/09	2009	Sarawak	m	Blood
RZ 197/09	2009	Selangor	m	Urine
RZ 207/09	2009	Selangor	m	Sputum
RZ 210/09	2009	Pahang	m	Blood
RZ 267/09	2009	Selangor	m	liver aspirate
RZ 367/09	2009	Johor	m	Blood
RZ 369/09	2009	Sarawak	f	Blood
RZ 370/09	2009	Sarawak	m	Blood
RZ 465/09	2009	Sarawak	m	Blood
RZ 466/09	2009	Sarawak	m	Blood
MZ 7/10	2010	Wilayah Persekutuan	m	Abscess
RZ 60/10	2010	Sarawak	f	Blood
RZ 61/10	2010	Sarawak	f	Blood
RZ 97/10	2010	Selangor	m	Pus
RZ 104/10	2010	Sarawak	f	Blood
RZ 154/10	2010	Johor	m	Blood
RZ 158/10	2010	Sarawak	m	Blood
RZ 161/10	2010	Johor	m	Blood
RZ 162/10	2010	Johor	f	Blood
RZ 164/10	2010	Sarawak	f	Pus
RZ 181/10	2010	Selangor	m	Blood
RZ 193/10	2010	Sarawak	m	Blood
RZ 205/10	2010	Pahang	m	Blood
RZ 206/10	2010	Pahang	m	Blood
RZ 207/10	2010	Pahang	m	Blood
RZ 208/10	2010	Pahang	m	Blood
RZ 209/10	2010	Pahang	m	Blood
RZ 210/10	2010	Selangor	m	Blood
RZ 229/10	2010	Sarawak	m	pus
RZ 236/10	2010	Perlis	m	blood
RZ 265/10	2010	Sarawak	m	blood
RZ 272/10	2010	Wilayah Persekutuan	m	blood
RZ 273/10	2010	Sarawak	m	blood
MZ 17/10	2010	Selangor	m	knee aspirate
MZ 24/10	2010	Pahang	m	pus

NA: not available.

**Table 2:** The MIC of antibiotics against 170 *Burkholderia pseudomallei* isolates.

Antibiotic	No. of isolates with the MIC (*µ*g/mL) of															MIC (*µ*g/mL)		%S
	≤0.25	0.38	0.5	0.75	1.0	1.5	2.0	3.0	4.0	6.0	8.0	12.0	16	128	≥256	MIC_50_	MIC_90_	
AMC						39	118	9	3						1	2.0	3.0	99.4
CAZ				1	11	75	65	15	2						1	1.5	2.0	99.4
CIP			7	23	42	56	28	10		2		1			1	1.5	2.0	42.3
CHL							1	14	37	81	31	4	1	1		6.0	8.0	96.5
SXT	18	14	19	16	28	24	34	10	5	2						1.0	2.0	90
DOX	1	10	11	26	48	48	20	4	1		1					1.0	2.0	99.4
IMI	48	114	7							1						0.38	0.38	99.4
MEM		2	12	98	49	8	1									0.75	1.0	100
TZP			2		50	99	16	1	2							1.5	2.0	100
SAM						1	47	99	20	1	1				1	3.0	4.0	99.4
TIG		5	15	6	13	32	39	36	19	4	1					1.5	4.0	64.7

AMC: amoxicillin/clavulanic acid; CAZ: ceftazidime; CIP: ciprofloxacin; CHL: chloramphenicol; SXT: trimethoprim-sulfamethoxazole; DOX: doxycycline; IMI: imipenem; MEM: meropenem; TZP: piperacillin/tazobactam; SAM: ampicillin/sulbactam; TIG: tigecycline.
